# Socioeconomic position and hysterectomy: a cross-cohort comparison of women in Australia and Great Britain

**DOI:** 10.1136/jech.2007.071001

**Published:** 2008-04-15

**Authors:** R Cooper, J Lucke, D A Lawlor, G Mishra, J-H Chang, S Ebrahim, D Kuh, A Dobson

**Affiliations:** 1 MRC National Survey of Health and Development, Department of Epidemiology and Public Health, University College London, London, UK; 2 School of Population Health, University of Queensland, Australia; 3 Department of Social Medicine, University of Bristol, UK; 4 Non-communicable Disease Epidemiology Unit, Department of Epidemiology and Population Health, London School of Hygiene and Tropical Medicine, London, UK

## Abstract

**Objectives:**

To examine the associations between indicators of socioeconomic position (SEP) and hysterectomy in two Australian and two British cohorts.

**Study population:**

Women participating in the Australian Longitudinal Study on Women’s Health (ALSWH), born 1921–1926 and 1946–1951, and two cohorts of British women, the British Women’s Heart and Health Study and the MRC National Survey of Health and Development, born at similar times (1920 to 1939 and 1946, respectively) and surveyed at similar ages to the ALSWH cohorts.

**Methods:**

Relative indices of inequality were derived for own and head of household occupational class, educational level attained and age at leaving school. Logistic regression was used to test the associations between these indicators of SEP and self-reported hysterectomy and/or oophorectomy.

**Results:**

Inverse associations between indicators of SEP and hysterectomy were found in both the Australian and British cohorts of women born in 1946 or later. There was also evidence of an inverse association between education and hysterectomy in the older Australian cohort. However, the associations in this older cohort were weaker than those found in the mid-aged Australian cohort. In the older British cohort, born in the 1920s and 1930s, little evidence of association between SEP in adulthood and hysterectomy was found.

**Conclusions:**

These results suggest that inverse associations between indicators of SEP and hysterectomy are stronger in younger than in older cohorts in both Australia and Great Britain. They provide further evidence of the dynamic nature of the association between indicators of SEP and hysterectomy.

It is well recognised that socioeconomic position (SEP) is associated with many health outcomes. However, these associations are not always consistently found across populations.^1^^–^3 It is therefore important to test whether there are differences in the socioeconomic differentials in health outcomes across populations in an effort to understand underlying mechanisms.^3^ ^4^

Socioeconomic differentials in hysterectomy, one of the most commonly performed surgical procedures on women in countries across the world, have been shown.^5^^–^7 However, associations between SEP and hysterectomy have not been consistently found and our comparison of the associations between lifetime SEP and hysterectomy in three cohorts of British women born in different decades of the twentieth century suggested that the nature of this association may vary by birth cohort.^2^ Consistent with most other studies that have found associations between indicators of SEP and hysterectomy, we found that among British women born in the 1940s and 1950s those of lower SEP had a greater risk of hysterectomy than those of higher SEP. However, in an older cohort of British women, born in the 1920s and 1930s, the converse was found and women from more deprived socioeconomic backgrounds had a reduced risk of hysterectomy compared with those from less deprived backgrounds. This suggests that socioeconomic differentials in hysterectomy may be dynamic, varying by birth cohort or period. Further, it suggests that socioeconomic differentials may be influenced by changes over time in access to medical care, women’s and doctors’ treatment preferences, the availability of alternative treatments and trends in characteristics such as fertility, oral contraceptive use and obesity.

Among women born between 1946 and 1951 and participating in the Australian Longitudinal Study on Women’s Health (ALSWH), indicators of SEP including having private health insurance and lower educational levels were found to be associated with increased risk of hysterectomy.^8^ However, variation in this association by birth cohort was not examined. When compared with British women of a similar age who were born at a similar time, this cohort of women living in Australia was found to have a higher prevalence of hysterectomy whether they were born in Australia or in Great Britain.^9^ This finding was attributed to differences in health service provision between countries.^9^ Given these differences, we cannot assume that the variation in socioeconomic differentials in hysterectomy by birth cohort found among British women will necessarily be seen among Australian women.

Using data from the two older ALSWH cohorts who were born at similar times to two of the British cohorts of women included in our original cross-cohort comparison we are able to extend our previous work, and examine whether there is also variation in the associations between SEP and hysterectomy by birth cohort among women resident in Australia, a country with higher rates of hysterectomy than Great Britain.

## METHODS

### Study populations

The ALSWH, alternatively known as Women’s Health Australia, consists of three cohorts of women resident in Australia, the oldest born between 1921 and 1926, the mid-aged cohort born between 1946 and 1951, and the youngest cohort born between 1973 and 1978. These cohorts of women were selected from the Health Insurance Commission database using stratified random sampling so that women living in rural and remote areas were selected at twice the rate of women in the same age group living in urban areas.^10^ This database covers all citizens and permanent residents of Australia, including refugees and immigrants. Baseline data were collected in 1996. Response rates to this first survey cannot be exactly specified, as some women selected for the sample may not have received the invitation, but an estimated 53–56% of the mid-aged women (n = 13 716), and 37–40% of the older women (n = 12 432) agreed to participate.^10^ The women in the ALSWH have been followed up regularly since baseline and retention rates for surveys 2 and 3 were 90% and 83%, respectively, for the mid-aged cohort and 89% and 90% for the older cohort once those who had died or were too ill to complete surveys had been excluded.^10^ Whether women had undergone a hysterectomy and/or oophorectomy was recorded at baseline as was information on socioeconomic circumstances. This information has been updated at subsequent data collections.

The British Women’s Heart and Health Study (BWHHS) is a cohort of 4286 women, born between 1920 and 1939, randomly selected from general practitioners’ lists in 23 towns across England, Scotland and Wales (the details of how these towns were selected is described elsewhere).^11^ Baseline data were collected between 1999 and 2001, when study members were 60–79 years old. At this time, gynaecological history and details of socioeconomic position across life were obtained retrospectively and details of contemporary socioeconomic circumstances were recorded.^11^ ^12^ Of 7173 women invited and eligible to participate in the baseline survey, information was obtained on 4286 (60%).

The MRC National Survey of Health and Development (NSHD) is a nationally representative cohort of 5362 men and women followed prospectively since their births across England, Scotland and Wales in March 1946. Information on health and social factors has been collected at time points across life.^13^ Questions on hysterectomy and oophorectomy were included at two home visits, at ages 43 and 53 years, and in a series of postal questionnaires sent annually between ages 47 and 54 years and at 57 years.^14^ Of the 2547 women in the original cohort 750 (29.4%) have not participated in a data collection since before reaching age 43 years.

The ALSWH received ethical approval from the University of Newcastle Ethics Committee (approval number: H-076-0795) and the University of Queensland Medical Research Ethics Committee (approval number: 200400224). Ethical approval for the most recent data collection, at age 53 years, of the NSHD was issued by North Thames Multi-centre Research Ethics Committee (MREC 98/1/121). Ethical approval for the NSHD women’s health postal questionnaires was issued by the Joint UCL/UCLH Committees on the Ethics of Human Research (Committee A) (ref 93/0057). The BWHHS received ethical approval from the 23 local research ethics committees covering the 23 towns from where the study participants were recruited and also from a multicentre research ethics committee.

The outcome in our study was self-report of hysterectomy and/or oophorectomy: by 1996 when women were aged 70–75 years in the oldest ALSWH cohort; by 2004 when the women were aged 53–58 years in the mid-aged ALSWH cohort; by 2003 when the women were aged 57 years in the NSHD; and at the time of the baseline survey (1999–2001) in the BWHHS when the women were aged 60–79 years.

Markers of SEP were selected if available in the Australian cohorts and at least one of the British cohorts. These were age at leaving full time education; highest level of educational qualification attained; own occupational class; and head of household occupational class.

Age at leaving full-time education and highest level of educational attainment were reported at baseline in the ALSWH and at age 26 years in the NSHD. Only the first of these measures of education was available in the BWHHS and this was collected at baseline. Age at leaving full-time education was categorised as shown in tables 1 and 2. In the ALSWH cohorts educational attainment was categorised as degree or higher; certificate or diploma; trade or apprentice; higher school certificate; school certificate; no formal qualification. In the NSHD, using as similar a categorisation as possible, it was grouped as university degree or higher; A levels or equivalent; O levels or equivalent; CSE, clerical course or equivalent; none.

**Table 1:** The associations (odds ratios (OR) and 95% confidence intervals (CI)) between indicators of socioeconomic position and hysterectomy and/or oophorectomy in the Australian Longitudinal Study on Women’s Health older and mid-aged cohorts (using samples with data on hysterectomy status and at least one measure of SEP)

	Australian Longitudinal Study on Women’s Health older cohort (born 1921–1926) (N = 12 792)			Australian Longitudinal Study on Women’s Health mid-aged cohort (born 1946–1951) (N = 14 078)		
	Total N	N (%) cases	OR (95% CI)	Total N	N (%) cases	OR (95% CI)
Own occupational class						
1. Professional and managerial	1451	501 (34.5)	1	3888	967 (24.9)	1
2. Para-professional	749	270 (36.1)	1.09 (0.83 to 1.42)	1222	380 (31.1)	1.38 (1.12 to 1.71)
3. Trades and administrative	2957	1074 (36.3)	1.08 (0.89 to 1.31)	3657	1120 (30.6)	1.33 (1.15 to 1.55)
4. Service and sales	1287	463 (36.0)	1.05 (0.84 to 1.32)	2123	718 (33.8)	1.55 (1.30 to 1.84)
5. Manual work/machine operators	1695	602 (35.5)	1.02 (0.82 to 1.26)	2034	695 (34.2)	1.57 (1.32 to 1.87)
Missing	*4653*	*1786 (38.4)*	*1.17 (0.98to 1.40)*	*1154*	*384 (33.3)*	*1.52 (1.22to 1.89)*
p Value*			0.95			<0.001
Head of household occupational class						
1. Professional and managerial	3689	1326 (35.9)	1	6908	1853 (26.8)	1
2. Para-professional	869	305 (35.1)	0.97 (0.78 to 1.22)	1193	404 (33.9)	1.38 (1.14 to 1.68)
3. Trades and administrative	2370	851 (35.9)	1.01 (0.86 to 1.18)	2867	923 (32.2)	1.28 (1.11 to 1.47)
4. Service and sales	791	288 (36.4)	1.03 (0.81 to 1.29)	1091	389 (35.7)	1.50 (1.23 to 1.83)
5. Manual work/machine operators	1582	585 (37.0)	1.05 (0.88 to 1.25)	1426	512 (35.9)	1.51 (1.26 to 1.80)
Missing	*3491*	*1341 (38.4)*	*1.12 (0.97to 1.28)*	*593*	*183 (30.9)*	*1.21 (0.91to 1.60)*
p Value*			0.60			<0.001
Age at leaving full-time education (years)						
⩾19	318	89 (28.0)	1	883	176 (19.9)	1
17–18	1644	574 (34.9)	1.39 (0.94 to 2.04)	3637	859 (23.6)	1.27 (0.96 to 1.69)
15–16	4911	1796 (36.6)	1.46 (1.01 to 2.11)	7885	2586 (32.8)	2.01 (1.54 to 2.62)
⩽14	5406	2048 (37.8)	1.57 (1.09 to 2.27)	1558	605 (38.8)	2.57 (1.90 to 3.46)
Missing	*513*	*189 (36.8)*	*1.47 (0.94to 2.30)*	*115*	*38 (33.0)*	*1.76 (0.88to 3.48)*
p Value*			0.01			<0.001
Highest educational qualification						
University degree or higher	439	129 (29.4)	1	1950	377 (19.3)	1
Certificate/diploma	908	304 (33.5)	1.27 (0.89 to 1.83)	2168	621 (28.6)	1.66 (1.33 to 2.06)
Trades and apprentice	426	155 (36.4)	1.44 (0.95 to 2.18)	489	147 (30.1)	1.78 (1.27 to 2.49)
High school certificate	1496	529 (35.4)	1.41 (1.00 to 1.98)	2344	657 (28.0)	1.59 (1.28 to 1.98)
School certificate	4633	1734 (37.4)	1.49 (1.09 to 2.04)	4427	1416 (32.0)	1.93 (1.59 to 2.34)
No formal qualifications	4209	1574 (37.4)	1.47 (1.07 to 2.02)	2551	1003 (39.3)	2.68 (2.17 to 3.29)
Missing	*681*	*271 (39.8)*	*1.69 (1.16to 2.47)*	*149*	*43 (28.9)*	*1.79 (0.99to 3.24)*
p Value*			0.02			<0.001

*From test for trend across categories, excluding missing category (italics).

Estimates are weighted to account for stratified sampling by area of residence.

**Table 2:** The associations (odds ratios (OR) and 95% confidence intervals (CI)) between indicators of socioeconomic position and hysterectomy and/or oophorectomy in two British cohorts: the British Women’s Heart and Health Study and the MRC National Survey of Health and Development (using samples with data on hysterectomy status and at least one measure of SEP)

	British Women’s Heart and Health Study (born 1920–1939) (N = 4286)			National Survey of Health and Development (born 1946) (N = 1782)		
	Total N	N (%) cases	Age-adjusted OR (95% CI)	Total N	N (%) cases	OR (95% CI)
Own occupational class						
I/II. Professional/managerial	788	197 (25.0)	1	588	129 (21.9)	1
IIInm. Skilled non-manual	1330	309 (23.2)	0.91 (0.74 to 1.11)	611	150 (24.6)	1.16 (0.89 to 1.51)
IIIm. Skilled manual	290	63 (21.7)	0.83 (0.60 to 1.15)	120	31 (25.8)	1.24 (0.79 to 1.95)
IV/V. Semi- or unskilled manual	823	184 (22.4)	0.86 (0.69 to 1.15)	322	85 (26.4)	1.28 (0.93 to 1.75)
Missing	*1055*	*168 (15.9)*	*0.57 (0.45to 0.72)*	*141*	*31 (22.0)*	*1.00 (0.64to 1.56)*
p Value*			0.21			0.12
Head of household occupational class						
I/II. Professional/managerial	923	205 (22.2)	1	873	207 (23.7)	1
IIInm. Skilled non-manual	665	143 (21.5)	0.96 (0.75 to 1.22)	229	51 (22.3)	0.92 (0.65 to 1.31)
IIIm. Skilled manual	1116	270 (24.2)	1.12 (0.91 to 1.37)	453	118 (26.1)	1.13 (0.87 to 1.47)
IV/V. Semi- or unskilled manual	1078	237 (22.0)	0.99 (0.80 to 1.22)	206	47 (22.8)	0.95 (0.66 to 1.36)
Missing	*504*	*66 (13.1)*	*0.53 (0.39to 0.71)*	*21*	*3 (14.3)*	*0.54 (0.16to 1.84)*
p Value*			0.79			0.73
Age at leaving full-time education (years)						
⩾19	334	72 (21.6)	1	62	10 (16.1)	1
17–18	379	103 (27.2)	1.36 (0.96 to 1.92)	483	104 (21.5)	1.43 (0.70 to 2.90)
15–16	1813	424 (23.4)	1.11 (0.84 to 1.47)	1155	286 (24.8)	1.71 (0.86 to 3.41)
⩽14	1438	286 (19.9)	0.90 (0.68 to 1.21)	–	–	–
Missing	*322*	*36 (11.2)*	*0.46 (0.30to 0.71)*	*82*	*26 (31.7)*	*2.41 (1.06to 5.49)*
p Value*			0.04			0.05
Highest educational qualification†						
University degree or higher	–	–	–	91	12 (13.2)	1
A level or equivalent	–	–	–	374	86 (23.0)	1.97 (1.02 to 3.78)
O level or equivalent	–	–	–	419	105 (25.1)	2.20 (1.15 to 4.20)
CSE, clerical course or equivalent	–	–	–	157	43 (27.4)	2.48 (1.23 to 5.01)
No formal qualifications	–	–	–	637	151 (23.7)	2.05 (1.08 to 3.85)
Missing	–	–	–	*104*	*29 (27.9)*	*2.55 (1.21to 5.35)*
p Value*			–			0.23

*From test for trend across categories, excluding missing category (italics).

†Not available in the British Women’s Heart and Health Study.

Own and head of household occupational class were collected at baseline in the ALSWH using the Australian Standard Classification of Occupations^15^ with categories grouped to be similar to the British ones. In the BWHHS measures of occupational class were recorded at baseline when participants were aged 60–79 years and in the NSHD at age 43 years (or at age 53, 36 or 26 years if missing at age 43 years). In both British cohorts occupational class was classified according to the Registrar General’s social classification groups as shown in table 2 for the first set of analyses and as I; II; III non-manual; III manual; IV; and V (with I representing those in professional occupations and V those in unskilled manual occupations) when calculating relative indices of inequality.

As in our previous study,^2^ we have used relative indices of inequality (RII). These enable direct comparison of the effect of SEP variables across cohorts because they take account of differences between cohorts in the proportions of women in the different categories of a socioeconomic variable.^16^ For each indicator of SEP a score between 0 (highest SEP) and 1 (lowest SEP) was assigned to each category based on the proportion of the population above the mid-point in that category. For example, if 10% of the population is in social class I, women in this group are represented by the range 0–0.1 and so are allocated the score 0.05 (ie, 0.1/2). If 20% of the population is in the next highest group, social class II, then this social class is allocated a score 0.20 (ie, 0.1+(0.2/2)) and so on. The RII is then obtained by regressing the outcome on each of these SEP scores and is directly interpretable for each SEP indicator used to compare women of the lowest SEP (1) with the highest SEP (0).

### Statistical analysis

The cumulative prevalence of hysterectomy and/or oophorectomy by age in each cohort was calculated and differences between Australian and British born women at specific ages in the ALSWH were examined.

As there was no information on timing of hysterectomy in the ALSWH, survival analysis was not possible; therefore, we used logistic regression to analyse proportions of women who had undergone hysterectomy and/or oophorectomy, obtaining odds ratios as the parameters of interest. Using logistic regression, we investigated the associations between each indicator of SEP and hysterectomy and/or oophorectomy in each of the four cohorts. In the first set of models, using the maximum available samples for each indicator of SEP, we entered indicators of SEP as categorical terms. In a second set of models, using samples with complete data on hysterectomy and all indicators of SEP, they were entered as continuous scores of inequality. In all analyses of the two Australian cohorts, estimates were weighted to account for the stratified sampling by area of residence.

## RESULTS

**Figure 1:**
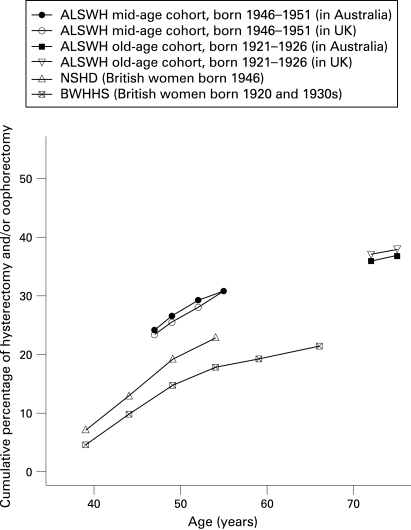
Cumulative prevalence of hysterectomy and/or oophorectomy by age in two Australian and two British cohorts.

Women in the two Australian cohorts were found to have a higher cumulative prevalence of hysterectomy than women in British cohorts of similar ages born at similar times (fig 1). A comparison of the cumulative prevalence of hysterectomy by place of birth in the two Australian cohorts found no significant differences by country of birth (fig 1) (test of difference in prevalence by country of birth in the mid-aged cohort, p = 0.86; and in the older cohort, p = 0.69)—women in the ALSWH cohorts born in Great Britain reported a similar prevalence of hysterectomy to those born in Australia. As there was also no significant variation in the association between SEP and hysterectomy by country of birth in the Australian cohorts (interaction between indicators of SEP and place of birth in the mid-aged cohort, all tests p>0.60; and in the older cohort, all tests p>0.40) it was not considered necessary to present results stratified by county of birth.

In the oldest ALSWH cohort, no association between own or head of household occupational class and hysterectomy was found (tables 1 and 3). However, in the mid-aged ALSWH cohort there were inverse associations between occupational class and hysterectomy, with those women in lower occupational classes experiencing greater odds of hysterectomy and/or oophorectomy than those of higher occupational classes. Similarly in the British cohorts, in the BWHHS (ie, the older cohort) there was no evidence of an association between occupational class and hysterectomy, whereas in the NSHD (a similar birth year and age to the ALSWH mid-aged cohort) there was weak evidence of an inverse association (tables 2 and 3).

**Table 3:** Age-adjusted associations (ORs and 95% CIs) between indicators of socioeconomic position and hysterectomy and/or oophorectomy in women born in the 1920s to 1940s resident in Australia and Great Britain, using relative indices of inequality (among women with complete data on hysterectomy and all indicators of socioeconomic position)

	Women resident in Australia		Women resident in Great Britain	
	ALSWH older cohort (born 1920–1926) N = 12 792; hysterectomy and/or oophorectomy = 4696	ALSWH mid-aged cohort (born 1946–1951) N = 14 078; hysterectomy and/or oophorectomy = 4264	BWHHS (born 1920–39) N = 3174; hysterectomy and/or oophorectomy = 739	NSHD (born 1946) N = 1518; hysterectomy and/or oophorectomy = 360
Own occupational class	1.00 (0.79 to 1.26)	1.79 (1.46 to 2.19)	0.83 (0.61 to 1.12)	1.54 (0.99 to 2.39)
Head of household occupational class	1.06 (0.85 to 1.32)	1.83 (1.49 to 2.25)	1.06 (0.78 to 1.43)	1.44 (0.93 to 2.23)
Age at leaving full-time education	1.26 (1.03 to 1.54)	2.74 (2.20 to 3.40)	0.90 (0.65 to 1.26)	1.87 (1.10 to 3.17)
Educational qualification	1.19 (0.97 to 1.44)	2.46 (2.02 to 3.00)	–	1.43 (0.92 to 2.22)

Australian estimates are weighted to account for stratified sampling by area of residence.

ALSWH, Australian Longitudinal Study on Women’s Health; BWHHS, British Women’s Heart and Health Study; NSHD, National Survey of Health and Development.

In both ALSWH cohorts there was an inverse association between educational level and hysterectomy—women with lower levels of education, as indicated by younger age at leaving education and less formal qualifications attained, experienced higher odds of hysterectomy than women with higher levels of education (tables 1 and 3). Comparison of results from RII models (table 3) suggest that the association between education and hysterectomy was stronger in the mid-aged than in the oldest ALSWH cohort. The association between education and hysterectomy was in the same inverse direction in the NSHD (tables 2 and 3). However, as previously found,^2^ ^5^ ^17^ there was evidence that the association between highest educational level attained and hysterectomy was non-linear, with the most basic level of qualification, rather than no qualifications, associated with the highest odds of hysterectomy and/or oophorectomy in the NSHD. In the BWHHS, the association between age at leaving education and hysterectomy was weak. However, there was some evidence to suggest any association of education with hysterectomy was in the opposite direction to that found in the other three cohorts (table 3).

## DISCUSSION

### Main findings

We have found inverse associations between indicators of SEP and hysterectomy in two cohorts of women born in the 1940s, one Australian and the other British. These associations, especially those between education and hysterectomy, were similar despite substantial differences in the prevalence of hysterectomy between the two countries. We also found inverse associations between some indicators of SEP, specifically education, and hysterectomy in an older Australian cohort, born in the 1920s. However, there was evidence to suggest that these associations were weaker than those found in the mid-aged Australian cohort. In the older British cohort, born in the 1920s and 1930s, little evidence of association between SEP in adulthood and hysterectomy was found. These results suggest that inverse associations between indicators of SEP and hysterectomy are stronger in younger than in older cohorts in both Australia and Great Britain.

### Comparison with other studies

Most existing studies of Australian women, including a study of women born between 1938 and 1948,^18^ that is, at a similar time to our mid-aged ALSWH cohort, have also found inverse associations between indicators of SEP and hysterectomy.^18^^–^22 However, findings from previous studies have not all been consistent and one study found no association between education or income and hysterectomy among South Australian women.^23^ Our study has important strengths as we have been able to compare results from Australian cohorts with British cohorts born at similar times who have been surveyed at similar ages using similar methods.

Our results for the ALSWH mid-aged cohort are consistent with findings from a previous study of this cohort,^8^ but the analyses have now been extended to include the older cohort. The results from new analyses of the British cohorts presented here differ slightly from those presented in our previous paper.^2^ This is due to the differences between papers in the approach to analyses used. These changes in analyses were necessary to ensure comparability between the British and Australian cohorts—the ALSWH cohorts do not have information on timing of hysterectomy, have collected slightly different measures of SEP and have not collected information on childhood socioeconomic circumstances. The differences between papers include the use of a different outcome definition (although results were similar to those presented when the analyses were re-run using the same outcome definition as used in the previous paper), the use of logistic regression rather than survival analysis, different categorisations of some SEP indicators including age at leaving school, differences in sample size and no stratification of analyses by age at hysterectomy. Despite these differences, the overall findings from the two papers in relation to the British cohorts are consistent as both papers show that lower SEP is associated with higher risk of hysterectomy in cohorts born in the 1940s but not in cohorts born earlier. Without the changes that have been made in this paper, comparisons with the Australian cohorts would have been subject to a greater number of limitations.

### Explanation of findings

There are a number of possible explanations of our finding of stronger inverse associations between indicators of SEP and hysterectomy in the two younger than in the two older cohorts. As discussed in our previous paper,^2^ there are likely to have been changes over time in factors such as access to medical care, attitudes of doctors, availability of oral contraceptives, changes in average family size and timing of birth, obesity and the availability of alternative treatments and patterns in uptake of these. Changes in these factors have been cited as reasons for variations in hysterectomy rates over time^24^ and may also explain changes in the socioeconomic differentials of hysterectomy over time. For example, timing of childbirth is likely to be more socially differentiated in younger than in older cohorts whereby hysterectomy may therefore also have become more socially differentiated. The similar findings in both countries, that is, inverse associations between SEP and hysterectomy are stronger in cohorts born from the mid-1940s onwards than in cohorts born earlier, suggests that the same factors may have operated in both countries even though there are marked differences in the prevalence of hysterectomy, which are most likely due to differences in medical and surgical practice.

Changes over time in the main reasons for hysterectomy and the treatment options for these could explain our findings. In earlier adulthood, the main reasons for hysterectomy are dysfunctional uterine bleeding, fibroids and other benign conditions.^25^ There are now alternative treatments available for many of these conditions and it is likely that they are more readily accessed by women of higher SEP.^26^ ^27^ Differential uptake of alternatives could partially explain the socioeconomic gradients in hysterectomy in our younger cohorts if women of lower SEP continue to use hysterectomy as a treatment while women of higher SEP select alternatives. For the older cohorts of women in our study such treatment alternatives would not have been available at the time when they were reporting these conditions (ie, in the 1970s) and consequently socioeconomic differences in hysterectomy may have been fewer. Further, as women age the reasons for hysterectomy change and decisions about treatment for the most prevalent gynaecological conditions reported in later adulthood (ie, cancer and prolapse) are based purely on medical considerations and involve less patient choice, with hysterectomy often being the only viable option.

It is possible that the differences between Australian birth cohorts are explained by changes with age in the association between SEP and hysterectomy. It has been shown in the NSHD and in other study populations that there are stronger associations between SEP and hysterectomies performed in earlier adulthood than in later adulthood.^2^ ^17^ ^27^ ^28^ Various explanations of this change in association with age have been proposed, including changes with age in the main reasons for hysterectomy and differences by SEP in the age at development and reporting of gynaecological symptoms. As timings of hysterectomies were not recorded in the ALSWH, it is not possible to test whether a similar change in association with age has been experienced in the Australian cohorts. It is therefore possible that any association between SEP and hysterectomies performed at earlier ages in the older ALSWH cohort has been diluted by the lack of association between SEP and hysterectomies performed for different reasons at later ages. However, in the older British cohort, the BWHHS, which has data on timing of hysterectomy, no changes were found in the associations between SEP and hysterectomy by age of procedure.^2^

A further consideration is whether education and occupation are able to measure the same aspects of SEP in women born in different birth cohorts. In both Great Britain and Australia, women’s access to education and their participation and role in the workforce has changed dramatically over time.^29^ ^30^ Irrespective of social standing, women born prior to the mid-1940s would have had greater difficulty in obtaining higher education and higher levels of occupation than those born more recently. Although the use of relative indices of inequality acts to standardise different measurements within a cohort and similar measurements across different cohorts, and should therefore deal with this issue to some extent, it may not completely mitigate different meanings of these exposures in different birth cohorts. Thus, the weaker associations in older women might reflect the inability of education and occupation to capture SEP as well in these cohorts as in the younger cohorts. Further, the greater workforce participation of women in the younger cohorts, particularly among lower socioeconomic groups, may have resulted in gynaecological symptoms such as heavy bleeding becoming less tolerable. This could have resulted in greater rates of referral and treatment among younger cohorts of women, especially those of lower SEP.

It is possible that the older cohorts of women are more highly selected groups and that the lack of socioeconomic gradients in these older cohorts is due to survival bias. However, this seems unlikely, as in both older cohorts it has already been shown that there are strong socioeconomic gradients in other health outcomes.^31^ ^32^

In our previous paper we acknowledged that differences in findings between cohorts could be an artefact of differences in study design and data collection methods. While this remains a possible explanation of the differences between the British cohorts, the fact that similar results and differences between cohorts are found in the Australian studies that employed the same design and data collection methods for both age groups makes this unlikely.

### Strengths and limitations

As discussed above, a major strength of this study is that it extends previous work by comparing associations not only across birth cohorts but also across two countries. These countries have similar ethnic and cultural backgrounds but different healthcare provision, as implied by the differences in prevalence of hysterectomy shown in this paper and a previous comparison between countries^9^ despite the fact that the prevalence of gynaecological morbidity is expected to be similar. Another strength of our analyses is that we were able to ensure that those performed were as similar as possible across cohorts and therefore comparable. Further, the same protocol was used for the two Australian cohorts and so we can be confident that differences in findings between birth cohorts are unlikely to be an artefact of differences in methods between cohorts.

As well as the aforementioned limitations, including the lack of information on timing of hysterectomies in the ALSWH, another limitation is the absence of information on reasons for hysterectomy in all cohorts. This is unfortunate as reasons for hysterectomy may have differed by age and between cohorts and so may have provided part of the explanation of our findings. A further limitation is that we ascertained hysterectomy status using self-reports. However, use of self-reported hysterectomy is unlikely to have introduced bias, as studies suggest that self-reports of hysterectomy and its timing are accurate.^33^ ^34^ Further, it has been found that valid results are obtained from analyses whether self-reported or hospital-recorded measures of hysterectomy are used.^35^

Bias may also have been introduced because of non-response, losses to follow-up and the exclusion of women with missing data. Although not all women invited to participate in the ALSWH did so, a comparison of the participants with census data confirmed that they were reasonably representative of the general population of women of the same age in Australia with the exception that women with tertiary education were overrepresented and some groups of immigrant women were underrepresented.^36^ In a comparison of responders and non-responders in the BWHHS it was found that non-responders were slightly younger and less likely to have a medical record report of ever having a stroke or diabetes, but had a similar prevalence of medical record-confirmed coronary heart disease and cancer.^11^ Further, the social class distribution of respondents was similar to that of women of the same age in the 1991 UK census.^11^ The NSHD was selected to be nationally representative and it has been shown that it remains so in most respects despite losses to follow-up.^37^ ^38^

Various factors not considered in these analyses—because they were not measured in comparable ways across cohorts or were not measured at appropriate time points in all cohorts—could potentially confound the relationship between SEP and hysterectomy. In other analyses of SEP and hysterectomy in the NSHD (R Cooper, unpublished PhD thesis, 2006)^5^ ^17^ the most likely confounders, parity, obesity and prior sterilisation, were controlled for and did not fully explain the associations found.

## CONCLUSIONS

This study provides further evidence of the dynamic nature of the association between indicators of SEP and hysterectomy and highlights the need to consider variation in association by birth cohort and where possible by age within cohorts. It also demonstrates the usefulness of cross-cohort comparisons in enabling us to elucidate the most likely explanations of socioeconomic differentials in health outcomes. The similarity in socioeconomic differentials in hysterectomy across countries despite the differences in health service provision, as indicated by differences in the prevalence of hysterectomy, suggests that factors other than those related to access to healthcare are likely to explain these differentials.

**What is already known on this subject**

A comparison of the association between socioeconomic position and hysterectomy in three cohorts of British women suggested that the nature of this association may vary by birth cohort. However, it is not known whether similar variations in association by birth cohort are found in other countries with different types of healthcare provision and rates of hysterectomy, such as Australia.

**What this study adds**

This study suggests that inverse associations between indicators of socioeconomic position and hysterectomy are stronger in younger than in older cohorts in both Australia and Great Britain. This similarity across countries, despite the differences in health service provision, as indicated by differences in the prevalence of hysterectomy, suggests that factors other than those related to access to healthcare are likely to explain these differentials.
